# Outcomes of CD19 CAR T in Transformed Indolent Lymphoma Compared to De Novo Aggressive Large B‐Cell Lymphoma

**DOI:** 10.1002/ajh.27548

**Published:** 2024-12-23

**Authors:** Swetha Kambhampati Thiruvengadam, Reid Merryman, Yan Wang, Charles Gaulin, Evandro Bezerra, Timothy Voorhees, Madhav R. Seshadri, Ayo Falade, Alma Habib, Amy A. Ayers, Megumi Bailey, Annette Brown, Neil Bailey, Krish Patel, Charalambos B. Andreadis, Adam S. Kittai, Caron Jacobson, Joycelynne Palmer, Stephen J. Forman, Loretta Nastoupil, Lihua E. Budde

**Affiliations:** ^1^ Department of Hematology and Hematopoietic Cell Transplantation City of Hope National Medical Center Duarte California USA; ^2^ Department of Hematologic Oncology Dana Farber Cancer Institute Boston Massachusetts USA; ^3^ Department of Computational and Quantitative Medicine, Division of Biostatistics City of Hope National Medical Center Duarte California USA; ^4^ Department of Lymphoma‐Myeloma, Division of Cancer Medicine MD Anderson Cancer Center Houston Texas USA; ^5^ Division of Hematology The Ohio State University Columbus Ohio USA; ^6^ Division of Hematology and Oncology University of California San Francisco San Francisco California USA; ^7^ Department of Medicine Mass General Brigham Salem Hospital Salem New Hampshire USA; ^8^ Center for Blood Disorders and Cellular Therapy Swedish Cancer Institute Seattle Washington USA; ^9^ Department of Hematology and Medical Oncology Icahn School of Medicine at Mount Sinai New York New York USA

**Keywords:** aLBCL, CAR T, DLBCL, transformed follicular lymphoma, transformed indolent lymphoma

## Abstract

Chimeric antigen receptor (CAR) T‐cell therapy has revolutionized treatment of aggressive large B‐cell lymphoma (aLBCL). Patients with transformed indolent non‐Hodgkin lymphoma (tiNHL) were included in key CAR trials, but outcomes of CAR for this distinct, historically high‐risk group are poorly understood. We conducted a multicenter retrospective study of 1182 patients with aLBCL receiving standard‐of‐care CAR T between 2017 and 2022, including 338 (29%) with tiNHL. Rates of grade ≥ 3 cytokine release syndrome (CRS) were similar between tiNHL and de novo cohorts (7% vs. 8%, *p* = 0.6), while grade ≥ 3 immune effector cell‐associated neurotoxicity syndrome was lower in tiNHL (21% vs. 27%, *p* = 0.02). Overall response rate was similar in both cohorts (83% vs. 81%, *p* = 0.3), while complete response rate was higher in tiNHL (67% vs. 59%, *p* = 0.017). With a median follow‐up of 22.3 months, the progression/relapse‐free (PFS) and overall survival (OS) were similar between the tiNHL and de novo cohorts (24‐month PFS 41% [95% CI: 35%–46%] vs. 38% [95% CI: 35%–42%]; 24‐month OS 58% [95% CI: 52%–63%] vs. 52% [95% CI: 48%–56%], respectively). After adjusting for key risk factors, there was a trend toward a lower hazard of disease progression, relapse or death post‐CAR for tiNHL patients compared to de novo aLBCL patients (HR: 0.84 [95% CI: 0.69–1.0], *p* = 0.07). Elevated LDH, advanced stage, prior bendamustine within 12 months of CAR, receipt of bridging therapy, CNS involvement, and ≥ 3 prior lines of therapy were each associated with inferior PFS. In conclusion, CAR T therapy is highly effective with an acceptable toxicity profile in patients with tiNHL.

## 1 Introduction

In recent decades, outcomes for patients with indolent non‐Hodgkin lymphoma (iNHL) have improved with studies showing similar overall survival (OS) to healthy age‐matched controls [1]; however, a subset of patients with iNHL (10%–30%) [2, 3] undergo transformation to an aggressive large B‐cell lymphoma (aLBCL) and represent a high‐risk patient subset with inferior OS [4]. Histologic transformation has been associated with chemotherapy resistance and inferior disease control, particularly for patients who received chemotherapy for their indolent lymphoma before transformation [2, 3, 5, 6, 7]. A large population‐based registry analysis demonstrated that patients with transformed follicular lymphoma (tFL) continue to have an inferior survival in the modern era compared to de novo aLBCL [8] with transformation associated with a substantially increased risk of death with lymphoma being the leading cause of death [9].

There is not a unique standard of care (SOC) for patients with transformed indolent NHL (tiNHL), and treatment guidelines were developed primarily based on studies for patients with de novo aLBCL. The treatment landscape for relapsed/refractory (R/R) aLBCL has changed dramatically with approvals for CD19 chimeric antigen receptor (CAR) T‐cell therapy, initially as third line and more recently as a second line for early relapsed or primary refractory patients. Patients with tiNHL comprised a small fraction (typically 10%–20%) of patients enrolled in the pivotal and registrational trials that led to the approvals of CD19 CAR T‐cell therapy in aLBCL [10, 11, 12, 13, 14, 15]. While some of these trials included subgroup analyses exploring outcomes of patients with tiNHL, these analyses were underpowered to draw conclusions with respect to efficacy of CAR T in tiNHL and importantly, none of these trials reported key safety endpoints separately among tiNHL patients. A larger study is needed to determine if there are unique safety or efficacy considerations for patients with tiNHL undergoing CAR T‐cell therapy.

Hence, we conducted a real‐world, multicenter retrospective study to evaluate the safety and efficacy of SOC CD19 CAR T‐cell therapy in adult patients with R/R tiNHL in comparison to de novo aLBCL.

## 2 Materials and Methods

### 2.1 Study Design and Patients

This is a multicenter retrospective study including six academic centers: City of Hope (COH), Dana Farber Cancer Institute, MD Anderson Cancer Center, Ohio State University, University of California San Francisco, and Swedish Cancer Institute. Eligibility criteria included: adult patients (≥ 18 years at time of CAR T infusion) with R/R aLBCL including diffuse large B‐cell lymphoma (DLBCL)/high‐grade B‐cell lymphoma (HGBCL) (both tiNHL and de novo) who received treatment with SOC CAR T‐cell therapy with infusion dates between 12/01/2017 and 10/31/2022. Patients with aLBCL transformed from FL (tFL), marginal zone lymphoma (tMZL), or Waldenstrom's Macroglobulinemia (tWM) were included, while patients with Richter transformation were excluded. Patients in complete response at time of CAR T infusion were excluded. Patients were followed post‐CAR T infusion through 06/30/2023, lost to follow‐up or death if it occurred before 06/30/2023. Study data were collected by the participating sites by chart review and managed using REDCap [16] electronic data capture tools hosted at COH. The study was approved by the Institutional Review Board at each site.

### 2.2 Treatments

Patients could have received any one of the three SOC CAR T‐cell products, axicabtagene ciloleucel (axi‐cel), tisagenlecleucel (tisa‐cel), and lisocabtagene maraleucel (liso‐cel). All patients received cyclophosphamide and fludarabine lymphodepletion (LD) therapy followed by CAR T‐cell infusion.

### 2.3 Key Definitions, Assessments, and Measurements

Bridging therapy was defined as any lymphoma‐specific therapy administered after leukapheresis and before conditioning chemotherapy. Pathologic diagnoses and molecular classification of patients with aLBCL by Hans algorithm were determined locally at each site [17]. Cytokine release syndrome (CRS) and immune effector cell‐associated neurotoxicity syndrome (ICANS) were graded according to American Society for Transplantation and Cellular Therapy (ASTCT) criteria [18]. Severe CRS and ICANS were defined as grade ≥ 3. Disease response was assessed using Lugano 2014 classification [19]. Best overall response rate (ORR) was defined as rate of patients who achieved the best response of complete response (CR) or partial response (PR) among patients who had disease response assessed post‐CAR T infusion. Duration of response (DOR) was defined as time from the onset of first documented response (CR/PR) to disease progression/relapse or death from any cause, whichever was observed first. Duration of complete response (DOCR) was defined as time from the onset of complete response (CR) to disease progression/relapse or death from any cause, whichever was observed first. OS was defined as the time from CAR T‐cell infusion to death due to any cause. Progression/relapse‐free survival (PFS) was defined as the time from CAR T‐cell infusion to disease progression/relapse (of either transformed or indolent histology in the tiNHL cohort) or death from any cause, whichever was observed first. Non‐relapse/progression mortality (NRM) was defined as death that was not preceded by disease relapse/progression. Patients were censored at the time of last contact if no event was observed.

### 2.4 Objectives and Endpoints

The primary objective was to evaluate the efficacy of SOC CD19 CAR T‐cell therapy in R/R tiNHL compared with de novo aLBCL, as measured by PFS, ORR, CR rate, DOR, DOCR, and OS. In addition, safety endpoints were evaluated including incidence and severity of CRS and ICANS, administration of tocilizumab and glucocorticoids, and rate of CAR T toxicity‐related intensive care unit (ICU) stay within the first 30 days post‐infusion. The secondary objective was to assess the difference in PFS post‐CAR T by bridging therapy and timing of prior bendamustine use.

In the tiNHL cohort, we explored the difference in PFS post‐CAR T by timing of transformation, prior treatment for underlying indolent disease before transformation, and type of indolent lymphoma, as well as the difference in best response by type of indolent lymphoma.

### 2.5 Statistical Considerations

Demographic and disease characteristics were summarized using descriptive statistics including median (range) for continuous variables and count (percentage) for categorical variables. Clopper–Pearson exact method was used to construct the 95% confidence intervals for ORR and CR rate among the patients who had disease response assessed post‐CAR T infusion. The Kaplan–Meier method was applied to estimate DOR, DOCR, PFS, and OS and log–log transformation method with Greenwood's formula for standard error was used to construct the 95% confidence intervals. Cumulative incidence of NRM was estimated using the Aalen–Johansen estimator, treating progression/relapse as the competing event.

As primary analysis, we fitted a multivariable Cox proportional hazards model to estimate the association between tiNHL cohort (vs. de novo) and PFS as measured by hazard ratio (HR) after adjusting for age at infusion (continuous, years), disease stage (per Ann Arbor) prior to CAR T (I–II/III–IV), ECOG performance score prior to CAR T (0–1/2–4), LDH > ULN prior to CAR T at leukapheresis (yes/no), > 1 extranodal site prior to CAR T (categorical, yes/no), ≥ 3 prior lines of therapy prior to CAR T (yes/no), prior bendamustine use timing relative to CAR T infusion (categorical, none/> 12 months/within 12 months), evidence of central nervous system (CNS) involvement of aLBCL prior to CAR T (yes/no), and receipt of bridging therapy (yes/no). These risk factors were pre‐specified per established International Prognostic Index (IPI) (age, disease stage, ECOG, LDH, and extranodal site) [20, 21] and a priori knowledge (heavily treated as defined by ≥ 3 prior lines of therapy, prior bendamustine use, CNS involvement, receipt of bridging therapy). In addition, Pearson's chi‐squared tests were performed to evaluate the difference in critical safety profile, ORR, and CR rate between tiNHL and de novo cohort. Given the differences in the biology of transformation based on the underlying indolent histology [22] and the comparatively rare incidence of tMZL and tWM, as a post hoc analysis, we excluded the patients in the tMZL and tWM subgroups and fitted a multivariable Cox proportional hazards model to estimate the association between tFL (vs de novo) and PFS as measured by HR after adjusting for the same key risk factor set as mentioned above.

As secondary analyses, we fitted an univariable Cox proportional hazards model to assess the association between PFS and prior bendamustine use (categorical, none/> 12 months/within 12 months prior to CAR T), and an univariable Cox proportional hazards model to assess the association between PFS post‐CAR T and receipt of bridging therapy.

As exploratory analyses, in the tiNHL cohort, we assessed whether PFS post‐CAR T differed by timing of transformation (concurrent/sequential), prior treatment for underlying indolent disease before transformation (yes/no), and type of indolent lymphoma (tFL/tMZL/tWM).

*P*‐values were two‐sided with a significance level of ≤ 0.05. Statistical analyses were performed in R version 4.3.0 (R Foundation for Statistical Computing, Vienna, Austria).

## 3 Results

### 3.1 Patient Characteristics

A total of 1182 patients were included in this retrospective study—338 (29%) with tiNHL and 844 (71%) with de novo aLBCL (Table 1). Most baseline characteristics were numerically similar in both cohorts. Among all patients, the median age at CAR T‐cell therapy was 64 (range: 18–89) years and 36% of patients were women. At the time of CAR T‐cell therapy, 934 (79%) patients had advanced‐stage disease (III–IV), 649 (55%) had an elevated LDH, 479 (41%) had more than one site of extranodal disease, and 135 (11%) had bulky disease (defined as ≥ 10 cm). In total, 580 patients (49%) received bridging therapy (chemotherapy in 346 patients [29%], radiation in 126 [11%], and steroids in 78 [7%]), with further details in Table S1. axi‐cel was the most frequently (77%) used CAR product followed by tisa‐cel (14%) and liso‐cel (9%).

**TABLE 1:** Baseline characteristics.

Characteristics	Overall, *N* = 1182^a^	De novo, *n* = 844^a^	tiNHL, *n* = 338^a^
Site name			
City of Hope	274 (23)	217 (26)	57 (17)
Dana Farber Cancer Institute	316 (27)	222 (26)	94 (28)
MD Anderson Cancer Center	316 (27)	223 (26)	93 (28)
The Ohio State University	162 (14)	107 (13)	55 (16)
University of California San Francisco	78 (7)	48 (6)	30 (9)
Swedish Cancer Institute	36 (3)	27 (3)	9 (3)
Age (years) at infusion	64 (18–89)	63 (18–89)	64 (33–88)
Age at infusion ≥ 60 years	768 (65)	533 (63)	235 (70)
Patient sex			
Female	424 (36)	301 (36)	123 (36)
Male	758 (64)	543 (64)	215 (64)
Lymphoma histology at infusion			
Diffuse large B cell lymphoma (DLBCL)	1062 (90)	768 (91)	294 (87)
High‐grade B‐cell lymphoma (HGBCL)	120 (10)	76 (9)	44 (13)
Disease stage (Ann Arbor) prior to CAR T			
I	69 (6)	59 (7)	10 (3)
II	159 (13)	114 (14)	45 (13)
III	225 (19)	143 (17)	82 (24)
IV	709 (60)	514 (61)	195 (58)
Unknown	20 (2)	14 (2)	6 (2)
Eastern Cooperative Oncology Group (ECOG) performance score prior to CAR T			
0	423 (36)	297 (35)	126 (37)
1	595 (50)	424 (50)	171 (51)
2	128 (11)	97 (11)	31 (9)
3	19 (2)	14 (2)	5 (1)
4	2 (0)	1 (0)	1 (0)
Unknown	15 (1)	11 (1)	4 (1)
LDH > ULN prior to CAR T (at leukapheresis)			
Yes	649 (55)	455 (54)	194 (57)
No	468 (40)	344 (41)	124 (37)
Unknown	65 (5)	45 (5)	20 (6)
> 1 extranodal site prior to CAR T			
Yes	479 (41)	352 (42)	127 (38)
No	697 (59)	486 (58)	211 (62)
Unknown	6 (1)	6 (1)	0 (0)
Had bulky disease (≥ 10 cm) prior to CAR T			
Yes	135 (11)	87 (10)	48 (14)
No	1027 (87)	743 (88)	284 (84)
Unknown	20 (2)	14 (2)	6 (2)
Evidence of CNS involvement of DLBCL/HGBCL prior to CAR T			
Yes	113 (10)	93 (11)	20 (6)
No	1068 (90)	750 (89)	318 (94)
Unknown	1 (0)	1 (0)	0 (0)
Number of prior lines of therapy before CAR T infusion (not including bridging)	3 (1–12)	3 (1–11)	3 (1–12)
Number of prior lines of therapy before CAR T infusion (not including bridging) (categorical)			
1	51 (4)	42 (5)	9 (3)
2	476 (40)	374 (44)	102 (30)
3	353 (30)	251 (30)	102 (30)
4+	302 (26)	177 (21)	125 (37)
≥ 3 prior lines of therapy (not including bridging)	655 (55)	428 (51)	227 (67)
Prior bendamustine use before apheresis (not including LD or bridging therapy)	206 (17)	68 (8)	138 (41)
Months from prior bendamustine^c^ to CAR T infusion	11 (0–129)	4 (0–83)	18 (1–129)
Prior bendamustine use^c^ timing relative to CAR T infusion			
Recent (within 12 months)	108 (9)	51 (6)	57 (17)
Remote (> 12 months)	98 (8)	17 (2)	81 (24)
None	976 (83)	776 (92)	200 (59)
Primary refractory disease prior to CAR T (refractory disease to initial therapy)	591 (50)	429 (51)	162 (48)
Refractory to most recent therapy prior to CAR T	915 (77)	657 (78)	258 (77)
No scan pre‐LD/CAR	1	0	1
Prior stem cell transplant			
Prior autologous HCT only	240 (20)	171 (20)	69 (20)
Prior allogeneic HCT only	17 (1)	11 (1)	6 (2)
Both prior autologous and allogeneic HCTs	8 (1)	8 (1)	0 (0)
None	917 (78)	654 (77)	263 (78)
Received prior CD19‐directed therapy	29 (2)	21 (2)	8 (2)
CAR T product name			
Axicabtagene ciloleucel	914 (77)	655 (78)	259 (77)
Tisagenlecleucel	166 (14)	117 (14)	49 (14)
Lisocabtagene maraleucel	102 (9)	72 (9)	30 (9)
Months from DLBCL/HGBCL diagnosis to CAR T infusion	14 (1–446)	15 (1–446)	12 (1–283)
Received bridging therapy	580 (49)	421 (50)	159 (47)
Chemotherapy based bridging therapy given	346 (29)	258 (31)	88 (26)
XRT bridging therapy given	126 (11)	94 (11)	32 (9)
Steroids bridging therapy given	78 (7)	53 (6)	25 (7)
Indolent lymphoma			
Follicular lymphoma			284 (84)
Marginal zone lymphoma			41 (12)
Waldenstrom's macroglobulinemia			13 (4)
Concurrent or sequential transformation			
Concurrent			86 (25)
Sequential			252 (75)
Months from indolent lymphoma diagnosis to transformation^b^			19 (0–434)
Any prior treatment for underlying indolent disease before transformation other than Rituximab (such as R‐CHOP, BR, R2)			213 (63)

^a^ Median (range); *n* (%).

^b^ One patient was diagnosed with DLBCL first, 7 months before the indolent component subsequently.

^c^ Prior bendamustine use: before apheresis (not including LD or bridging therapy).

Abbreviations: BR, bendamustine‐rituximab; CAR T, chimeric antigen receptor T‐cell; CNS, central nervous system; HCT, hematopoietic cell transplantation; LD, lymphodepleting; R CHOP, rituximab, cyclophosphamide, vincristine, doxorubicin, prednisone; R2, lenalidomide, rituximab; XRT, radiation.

Compared to patients in de novo cohort, patients with tiNHL were more heavily pre‐treated—67% vs. 51% had received ≥ 3 prior lines of therapy before CAR, more likely to have HGBCL (13% vs. 9%), but were less likely to have CNS involvement (6% vs. 11%). Prior bendamustine treatment (before apheresis not including LD or bridging therapy) was more common among patients with tiNHL (41% vs. 8%), and tended to be more remote (median 18 months [range: 1–129] vs. 4 months [range: 0–83] before CAR infusion). Seventeen percent of patients in the tiNHL and 6% in the de novo cohort received bendamustine within 12 months of CAR T‐cell infusion.

Among patients with tiNHL, 284 (84%) had tFL, 41 (12%) tMZL, and 13 (4%) tWM. Transformation was detected at the time of initial indolent lymphoma diagnosis (concurrent) in 25% of patients, while transformation occurred later (sequential) for the remaining 75% of patients. Two hundred and thirteen (63%) patients with tiNHL received chemotherapy or lenalidomide‐based treatment for an underlying indolent lymphoma before transformation. The median time from indolent lymphoma diagnosis to transformation was 19 (range: 0–434) months (Table 1).

### 3.2 Safety

Any grade CRS was observed in 79% of tiNHL patients and 84% of de novo aLBCL patients with the median time between CAR T infusion and first CRS onset of 3 (range: 0–53) days and 2 (range: 0–23) days, respectively (Table 2). Patients in the tiNHL cohort were less likely to experience CRS within the first 2 days post‐CAR T (36% vs. 42%, *p* = 0.031). Rates of grade ≥ 3 CRS were similar in the tiNHL and de novo cohorts (7% vs. 8%, *p* = 0.6). Patients in the tiNHL cohort were less likely to receive tocilizumab for CRS than those in the de novo cohort [50% vs. 61%, *p* < 0.001], while rates of glucocorticoid use for CRS management were similar (26% vs. 28%, *p* = 0.5). Within the tiNHL cohort, any grade CRS was observed in 81% of patients with tFL, 71% of tMZL, and 69% of tWM, while grade ≥ 3 CRS was observed in 6%, 15%, and 0%, respectively (Table S2).

**TABLE 2:** Safety profile.

Characteristics	Overall, *N* = 1182^a^	De novo, *n* = 844^a^	tiNHL, *n* = 338^a^	*p*‐value^b^
CRS any grade	974 (82)	707 (84)	267 (79)	0.051
Days between infusion and first CRS onset	3 (0–53)	2 (0–23)	3 (0–53)	0.016
CRS onset within the first 2 days post CAR T infusion	477 (40)	357 (42)	120 (36)	0.031
CRS ≥ Grade 3	91 (8)	67 (8)	24 (7)	0.6
Maximum CRS grade (per ASTCT consensus grading)				
Grade 1	489 (41)	355 (42)	134 (40)	
Grade 2	394 (33)	285 (34)	109 (32)	
Grade 3	59 (5)	44 (5)	15 (4)	
Grade 4	31 (3)	23 (3)	8 (2)	
Grade 5	1 (0)	0 (0)	1 (0)	
No CRS	208 (18)	137 (16)	71 (21)	
Tocilizumab given for CRS	686 (58)	516 (61)	170 (50)	< 0.001
Glucocorticoids were given for CRS	325 (27)	237 (28)	88 (26)	0.5
ICANS any grade	580 (49)	439 (52)	141 (42)	0.001
Days between infusion and first ICANS onset	6 (0–308)	5 (0–308)	6 (0–38)	0.4
ICANS ≥ Grade 3	298 (25)	228 (27)	70 (21)	0.024
Maximum ICANS grade (per ASTCT consensus grading)				
Grade 1	137 (12)	111 (13)	26 (8)	
Grade 2	145 (12)	100 (12)	45 (13)	
Grade 3	236 (20)	179 (21)	57 (17)	
Grade 4	60 (5)	47 (6)	13 (4)	
Grade 5	2 (0)	2 (0)	0 (0)	
No ICANS	602 (51)	405 (48)	197 (58)	
Glucocorticoids were given for ICANS	489 (41)	369 (44)	120 (36)	0.010
CAR T toxicity‐related ICU stay within the first 30 days post‐infusion	159 (13)	120 (14)	39 (12)	0.2

^a^ *n* (%); median (range).

^b^ Pearson's chi‐squared test; Wilcoxon's rank‐sum test.

Abbreviations: ASTCT, American Society for Transplantation and Cellular Therapy; CRS, cytokine release syndrome, ICANS, immune effector cell‐associated neurotoxicity syndrome.

Any grade ICANS were observed in fewer tiNHL patients compared to de novo (42% vs. 52%, *p* = 0.001) with the median time between CAR T infusion and first ICANS onset of 5 (range: 0–308) days and 6 (range: 0–38) days, respectively. Grade ≥ 3 ICANS was lower in the tiNHL cohort compared to the de novo cohort (21% vs. 27%, *p* = 0.024). Glucocorticoid use for ICANS was lower among patients in the tiNHL cohort compared to the de novo cohort (36% vs. 44%, *p* = 0.010). Within the tiNHL cohort, any grade ICANS was observed in 41% of patients with tFL, 44% of tMZL, and 46% of tWM, while grade ≥ 3 ICANS was observed in 20%, 22%, and 23%, respectively.

Within the first 30 days post‐CAR T infusion, rates of CAR T toxicity‐related ICU admissions were similar between the tiNHL and de novo cohorts (12% vs. 14%, *p* = 0.2). Within the tiNHL cohort, CAR T toxicity‐related ICU admissions occurred in 11% of tFL, 17% of tMZL, and 15% of tWM patients.

### 3.3 Disease Response

Best ORR was similar for patients with tiNHL and de novo aLBCL (83% [95% CI: 79%–87%] vs. 81% [95% CI: 78%–83%], *p* = 0.3), while CR rate was higher among patients with tiNHL (67% [95% CI: 62%–72%] vs. 59% [95% CI: 56%–63%], *p* = 0.017) (Table S3, Figure S1A). Among 931 responders, the median time from CAR T infusion to first documented response was 1.0 (range: 0.5–4.1) months in the tiNHL cohort and 1.0 (range: 0.6–9.3) months in the de novo cohort. Within the tiNHL cohort, ORR and CR rate for patients with tFL were 84% (95% CI: 79%–88%) and 68% (95% CI: 62%–73%) respectively, 82% (95% CI: 67%–93%) and 65% (95% CI: 48%–79%) for tMZL, and 77% (95% CI: 46%–95%) and 62% (95% CI: 32%–86%) for tWM (Figure S1B).

**FIGURE 1:**
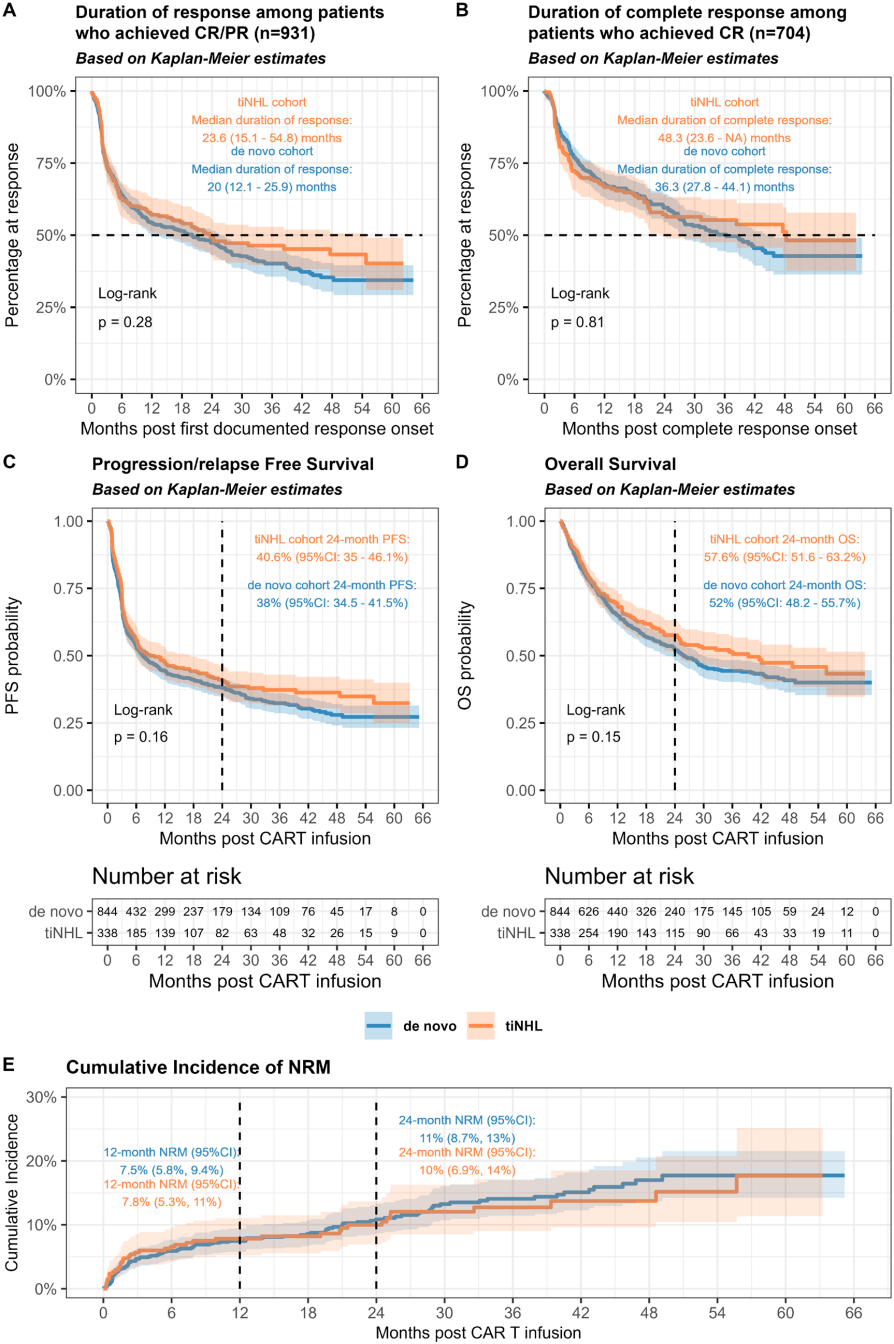
Duration of response and survival outcomes. (A) Duration of overall response, (B) duration of complete response, (C) progression/relapse‐free survival, (D) overall survival, (E) cumulative incidence of non‐relapse/progression mortality (NRM). [Color figure can be viewed at wileyonlinelibrary.com]

The median DOR was 23.6 (95% CI: 15.1–54.8) months in the tiNHL cohort and 20.0 (95% CI: 12.1–25.9) months in the de novo cohort (*p* = 0.28) (Figure 1A). Among 704 patients who achieved CR, the median duration of CR was 48.3 (95% CI: 23.6–not reached) months in tiNHL cohort and 36.3 (95% CI: 27.8–44.1) months in de novo cohort (*p* = 0.81) (Figure 1B).

### 3.4 Survival

With a median follow‐up of 13.0 (range: 0.03–65.2) months post CAR T infusion among all patients and 22.3 (range: 0.03–65.2) months among survivors, the median PFS was 8.8 (95% CI: 6.1–17.3) months in the tiNHL cohort and 7.3 (95% CI: 6.0–9.8) months in the de novo cohort, with 24‐month PFS of 40.6% (95% CI: 35.0%–46.1%) and 38.0% (95% CI: 34.5%–41.5%), respectively (*p* = 0.16) (Figure 1C) Within the tiNHL cohort, the 12‐month PFS was 49.4% (95% CI: 43.3%–55.1%) in tFL, 43.9% (95% CI: 28.6%–58.2%) in tMZL, and 30.8% (95% CI: 9.5%–55.4%) in tWM (Figure S3C).

During follow‐up, 160 patients (50%) in the tiNHL cohort and 425 patients (53%) in the de novo cohort experienced disease progression/relapse (Table S4). Among 82 patients in the tiNHL cohort who had a biopsy collected at the time of post‐CAR relapse/progression, 72 (88%) had aLBCL present, 8 (10%) had indolent lymphoma present, and 2 (2%) had both histologies present. The median time from infusion to progression or relapse was 3.1 (range: 0.3–24.0) months for patients who relapsed with aLBCL and 10.2 (range: 1.6–22.2) months for patients with an indolent lymphoma relapse.

The median OS was 39.4 (95% CI: 24.9–not reached) months in the tiNHL cohort and 25.8 (95% CI: 22.1–29.7) months in the de novo cohort, with 24‐month OS of 57.6% (95% CI: 51.6%–63.2%) and 52% (95% CI: 48.2%–55.7%) respectively (*p* = 0.15) (Figure 1D).

Forty‐three percent (*n* = 144) patients in the tiNHL cohort and 47% (*n* = 393) in the de novo cohort died during the study follow‐up, with 10% (*n* = 14) and 8% (*n* = 31) deaths considered CAR T therapy‐related, respectively (Table S4). The cumulative incidence of NRM at 12 months was 7.8% (95% CI: 5.3%–11%) in the tiNHL cohort and 7.5% (95% CI: 5.8%–9.4%) in the de novo cohort, and 10% (95% CI: 6.9%–14%) and 11% (95% CI: 8.7%–13%) at 24‐month post‐CAR T infusion, respectively (Figure 1E).

### 3.5 Baseline Risk Factors for PFS Post‐CAR T

After adjusting for key clinical factors at time of CAR T infusion, there was a trend toward a lower hazard of disease progression, relapse, or death (adjusted HR [aHR]: 0.84 [95% CI: 0.69–1.0], *p* = 0.07) among tiNHL cohort compared to de novo cohort (Table 3). In a post hoc analysis limited to patients with either tFL or de novo aLBCL, the hazard of disease progression, relapse, or death was significantly lower for patients with tFL compared to those with de novo aLBCL (aHR: 0.81 [95% CI: 0.66–1.0], *p* = 0.049) (Table S5). The regression model suggested that other factors associated with worse PFS included elevated LDH (aHR: 1.7 [95% CI: 1.5–2.0], *p* < 0.001), advanced disease stage (aHR: 1.4 [95% CI: 1.1–1.8], *p* = 0.002), ≥ 3 prior lines of therapy (aHR: 1.2 [95% CI: 1.0–1.4], *p* = 0.043), bendamustine use within 12 months prior to CAR T (aHR: 1.5 [95% CI: 1.1–1.9], *p* = 0.003), CNS involvement (aHR: 1.3 [95% CI: 1.0–1.6], *p* = 0.036), and receipt of bridging therapy (aHR: 1.4 [95% CI: 1.2–1.6], *p* < 0.001).

**TABLE 3:** Multivariable Cox proportional hazards model for progression/relapse‐free survival.

Variable	Group	*N* ^a^	Number of events	Adjusted HR (95% CI)	Forest plot: Adjusted HR (95% CI)	*p*‐value
Cohort	De novo	769	483	1 (Ref.)	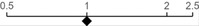	
	tiNHL	310	182	0.8 (0.7, 1)	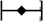	0.07
Age (years) at infusion	Every additional 1 year older			1 (0.99, 1)	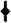	0.297
Disease stage (Ann Arbor) prior to CAR T	I–II	203	104	1 (Ref.)	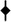	
	III–IV	876	561	1.4 (1.1, 1.8)	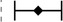	0.002
ECOG Performance score prior to CAR T	0–1	940	571	1 (Ref.)	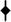	
	2–4	139	94	1 (0.81, 1.3)	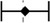	0.871
LDH > ULN prior to CAR T (at leukapheresis)	No	449	223	1 (Ref.)	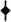	
	Yes	630	442	1.7 (1.5, 2)	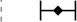	< 0.001
> 1 extranodal site prior to CAR T	No	644	379	1 (Ref.)	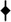	
	Yes	435	286	1 (0.88, 1.2)	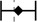	0.635
Prior lines of therapy before CAR T infusion (not including bridging) ≥ 3	No	469	265	1 (Ref.)	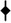	
	Yes	610	400	1.2 (1, 1.4)	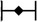	0.043
Prior bendamustine use^b^ timing relative to CAR T infusion	None	892	541	1 (Ref.)	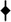	
	Remote (> 12 months)	90	49	0. 9 (0.6, 1.2)	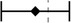	0.461
	Recent (within 12 months)	97	75	1.5 (1.1, 1.9)	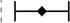	0.003
Evidence of CNS involvement of aLBCL prior to CAR T	No	972	580	1 (Ref.)	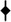	
	Yes	107	85	1.3 (1, 1.6)	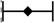	0.036
Receipt of bridging therapy	No	538	300	1 (Ref.)	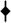	
	Yes	541	365	1.4 (1.2, 1.6)	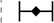	< 0.001

^a^ One‐hundred and three out of 1182 patients had unknown disease stage, ECOG, LDH, extranodal site, and/or CNS involvement of aLBCL prior to CAR T and thus were excluded from the multivariable model.

^b^ Prior bendamustine use: before apheresis (not including LD or bridging therapy).

Abbreviations: CAR T, chimeric antigen receptor T‐cell; CNS, central nervous system; ECOG, Eastern Cooperative Oncology Group; LDH, lactate dehydrogenase; ULN, upper limit of normal.

As secondary analyses, we found that when compared to patients with no prior bendamustine use, PFS appears to be similar in the patients with remote (beyond 12 months prior) bendamustine use (HR: 0.9 [95% CI: 0.6–1.1], *p* = 0.255), while PFS was inferior in patients with recent (within 12 months prior) bendamustine use (HR: 1.7 [95% CI: 1.4–2.1], *p* < 0.001) (Figure S2A). In addition, receipt of bridging therapy was associated with inferior PFS post‐CAR T (HR: 1.5 [95% CI: 1.3–1.8], *p* < 0.001) (Figure S2B).

An exploratory analysis among the tiNHL cohort suggested that patients with sequential transformation appear to have better PFS compared to those with concurrent transformation (*p* = 0.016) (Figure S3A). In contrast, prior treatment for indolent lymphoma before transformation did not appear to impact PFS post‐CAR T infusion (*p* = 0.24) (Figure S3B). Finally, PFS appeared to be similar for different subtypes of tiNHL, but the analysis was limited by low numbers of patients with tMZL and tWM (*p* = 0.34) (Figure S3C). Kinetics of paraprotein change in Waldenstrom patients after CAR T is shown in Figure S4.

## 4 Discussion

This is the largest study of CD19 CAR T‐cell therapy for patients with tiNHL, suggesting that CAR T is a highly effective and safe treatment option for tiNHL patients, with noticeable differences in outcomes when compared to de novo aLBCL patients.

With respect to safety, we observed lower rates of grade ≥ 3 ICANS and reduced use of glucocorticoids for ICANS management for patients with tiNHL. Several factors might explain the differences in ICANS rates. ICANS frequencies appear to be higher among patients with aggressive lymphomas (DLBCL, MCL) compared to indolent lymphomas [10, 11, 12, 13, 14, 15, 23, 24, 25]. Patients with tiNHL may have an intermediate ICANS risk between de novo aLBCL and indolent lymphomas. Additionally, patients with de novo aLBCL were more likely to have rapid onset CRS (occurring within the first 2 days), which has previously been associated with an increased risk of ICANS [26]. Furthermore, de novo aLBCL patients were more likely to receive tocilizumab for CRS management, which has been associated with ICANS risk [26], although it is likely that this reflects differences in underlying CRS that prompted tocilizumab use rather than direct neurotoxic effects of tocilizumab.

We also found potential differences in CAR T‐cell efficacy for patients with tiNHL. Patients with tiNHL had higher CR rates compared to de novo aLBCL (67% vs. 59%), a finding suggested in phase II trials of axi‐cel, tisa‐cel, and lis‐cel [10, 12, 15]. In an uncontrolled comparison, there was no difference in PFS between patients with tiNHL and de novo aLBCL; however, the tiNHL cohort had more frequent high‐risk features, including more prior lines of therapy and more frequent treatment with bendamustine before T‐cell collection. After accounting for key differences in a multivariable analysis, it appears that patients with tiNHL may have a lower risk of death or relapse/progression compared to those with de novo aLBCL. These findings also align with small subset analyses from Phase 2 trials of tisa‐cel and liso‐cel, which suggested that PFS may be improved for patients with tFL [12, 15] and a recent smaller DESCAR‐T registry study [27]. In addition, this study provides useful information about patterns of disease control for patients with tiNHL who have a heightened risk of both indolent and aggressive lymphoma recurrence. Among 82 (51%) patients with tiNHL who had a biopsy at relapse, nearly 90% had a relapse of their aggressive lymphoma (at a median 3 months after CAR T infusion), while only 10% of tiNHL patients relapsed with indolent lymphoma (at median 10 months after CAR T infusion). This may be influenced by the short median follow‐up where relapse of the aggressive histology is more likely than the indolent component.

In our multivariable regression analysis, baseline characteristics such as elevated LDH, advanced stage, ≥ 3 prior lines of therapy, prior bendamustine use within 12 months of CAR T, CNS involvement, and receipt of bridging therapy were statistically significantly associated with inferior PFS outcomes in the overall cohort. These prognostic biomarkers are consistent with those identified in prior studies evaluating predictive factors of early progression after CAR T‐cell therapy in R/R aLBCL [28, 29, 30, 31]. Of note, in our analysis, although the *p*‐values for ≥ 3 prior lines of therapy and CNS involvement were less than 0.05, indicating statistical significance, the 95% confidence intervals included 1. This suggests that the associations between PFS and these 2 risk factors may not be robust or could be influenced by variability in the data. Bendamustine use prior to CAR T was reported as a risk factor of clinical outcomes in previous studies [29, 30] but the timing of prior bendamustine use (before apheresis not including LD or bridging therapy) relative to CAR T was not fully understood. To our knowledge, this is the largest published study assessing the association between prior bendamustine timing and PFS post‐CAR in R/R aLBCL, and builds on prior studies suggesting that recent bendamustine exposure is associated with inferior PFS [29].

For the purposes of our primary analyses, we combined all patients with tiNHL together into a single cohort; however, there may be important differences in CAR T‐cell safety and efficacy depending on the underlying indolent lymphoma subtype. Small numbers of patients with tMZL and tWM preclude adequately powered comparisons between the different tiNHL subgroups. The post hoc analysis suggests that tFL has favorable outcomes compared to de novo aLBCL. The improvement of outcomes when comparing tFL alone (as opposed to tiNHL overall) with de novo DLBCL suggests that transformation of FL is biologically distinct from transformation of MZL and WM (Table 3 and Table S5). The preliminary patterns observed in this study, such as numerically inferior response rates and 1‐year PFS in the tMZL and tWM cohort and numerically higher grade ≥ 3 CRS in the tMZL cohort compared to the tFL subgroup (Table S2, Figures S1B,S3C), are hypothesis‐generating and should be investigated in larger studies in the future.

While this study represents the largest analysis of outcomes of CAR therapy for patients with tiNHL, several limitations extend beyond the retrospective nature of the study. The median follow‐up of nearly 2 years, is long enough to identify most aggressive lymphoma relapses, but longer follow‐up is warranted, particularly to capture more delayed relapses that can be observed with indolent NHLs. ASTCT consensus grading was used for CRS and ICANS at all centers, but some patients were treated before publication of this grading system, requiring retrospective assignment of CRS or ICANS grade. In addition, we acknowledge differences in CRS and ICANS management at individual centers could impact timing and severity of CRS and ICANS. Similar to other studies [28, 29, 30], we show that use of bridging therapy and prior bendamustine exposure are associated with worse outcomes with CAR, but we acknowledge the potential for confounders and interaction effects with other factors. Further studies that are specifically designed to address the impacts of bridging and the timing of prior bendamustine exposure relative to CAR T are needed. Finally, we collected key clinical variables associated with efficacy and safety outcomes for CAR T‐cell therapy, but we were not able to include laboratory assessments (i.e., ferritin, C‐reactive protein) or imaging features (i.e., total tumor metabolic volume) that have been associated with CRS, ICANS, response rates, and PFS in previous CAR T‐cell trials.

Despite these limitations, this study has multiple implications for both patient care and future clinical investigation. Prior studies suggest that patients with tiNHL are likely to have inferior disease control with chemotherapy, particularly patients who received treatment before transformation [2, 7]. Key trials testing CD19 CAR T‐cell therapy as initial treatment for DLBCL such as ZUMA‐12 [32] and ZUMA‐23 [33] exclude tiNHL patients who have received prior treatment for their indolent lymphoma. Our study suggests that these patients would be good candidates for clinical trials that move CAR T‐cell therapy to earlier lines of therapy, both because of poor expected outcomes with chemoimmunotherapy and because of excellent outcomes with CAR T cells.

In conclusion, CD19 CAR T‐cell therapy is a highly effective treatment option with no new safety signals for tiNHL, a disease that was underrepresented in prior pivotal CAR T trials and historically associated with inferior outcomes with conventional therapies. In comparison to patients with de novo aLBCL, patients with tiNHL appear to have lower rates of ICANS and higher complete response rates with CAR therapy. After accounting for other key variables, PFS may also be superior for patients with tiNHL compared to those with de novo aLBCL. Longer follow‐up is needed to confirm these findings, particularly given the heightened risk of delayed indolent lymphoma relapse for patients with tiNHL. Additional studies are needed to identify predictive biomarkers of relapse after CAR T in tiNHL patients and to better understand potential differences in less common subtypes of tiNHL.

## Author Contributions

S.K.T., R.M., and E.B. designed the study. Y.W. built and managed the study database. S.K.T., R.M,. C.G., E.B., T.V., M.R.S., A.F., A.H., A.A.A., M.B., A.B., N.B., K.P., C.B.A., A.S.K., and C.J. collected the data. Y.W., J.P., S.K., R.M., and E.B. contributed to the analysis plan, performed the data analysis and provided the interpretation. S.K., R.M., Y.W., and E.B. wrote the manuscript. All authors contributed to reviewing and editing the manuscript.

## Ethics Statement

The authors have nothing to report.

## Consent

The authors have nothing to report.

## Conflicts of Interest

Swetha Kambhampati Thiruvengadam: Research funding—Genentech, Genmab, ADC‐Therapeutics, Ipsen. Consulting—Abbvie, Advisory Board—Ipsen. Reid Merryman: Advisory board—Genmab, Adaptive Biotechnologies, Bristol Myers Squibb, Abbvie, Inteillia, Epizyme. Consulting—AlphaSights. Institutional research funding—Merck, Bristol Myers Squibb, Genmab, Genentech/Roche. Charles Gaulin: Honoraria‐DeciBio. Advisory Board—ADC Therapeutics, Sanofi. Consulting—Novartis, Kyverna and Kite. Timothy Voorhees: Advisory Board—Genmab/AbbVie. Consultancy—Novartis, Recordati, Genmab. Research Funding—Kite, Viracta, Incyte/Morphosys, Genmab/AbbVie, Recordati. Madhav R. Seshadri: Institutional research funding—Genentech, Eli Lilly, Kyverna Therapeutics. Consulting—Kite Pharma, BeiGene, Abbvie, AstraZeneca. Charalambos B. Andreadis: Consulting—BMS, Kite/Gilead, Novartis. Research Funding: Merck, Genentech. Adam S. Kittai: Research funding—AstraZeneca, BeiGgene. Advisory Board—Abbvie, AstraZeneca, BeiGene, BMS, Eli‐Lilly, Janssen, KITE. Speakers Bureau—BeiGene. Krish Patel: Research funding (to institution)—Abbvie, AstraZeneca, BMS/Celgene, Cargo, Caribou, Century Therapeutics, CRISPR Therapeutics, Fate Therapeutics, Genentech/Roche, Janssen, Kite, Loxo/Lilly, Merck, Nurix Therapeutics, Pharmacyclics, Pfizer, Sana, Xencor. Consulting‐Abbvie, Adaptive Biotechnologies, ADC Therapeutics, AstraZeneca, Beigene, BMS/Celgene, Caribou, Genentech/Roche, Janssen, Kite, Loxo/Lilly, Merck, Nurix Therapeutics, Pharmacyclics, Pfizer, Sana, Xencor. Caron Jacobson: Consultancy—Miltenyi Biotec, Morphosys, Ipsen, Daiichi‐Sankyo, ImmPACT Bio, Instill Bio, Caribou Bio, Abintus Bio, AstraZeneca, ADC Therapeutics, Abbvie, BMS/Celgene, Kite/Gilead, Novartis, Epizyme, Bluebird Bio, Humanigen, Nkarta, Precision BioSciences. Loretta Nastoupil: Honoraria—AbbVie, ADC Therapeutics, BMS/Celgene, Regeneron, AstraZeneca. Research funding‐Daiichi Sankyo, Genentech, Genmab, Gilead/Kite, Janssen, Merck, Novartis, Takeda, Gilead Sciences/Kite. Lihua E. Budde: Consulting—Genentech, MustangBio, Roche, AstraZeneca, Amgen, Merck, ADC Therapeutics. The remaining authors declared no conflicts of interest.

## Supporting information


**Data S1.** Supporting Information.

## Data Availability

The data that support the findings of this study are available from the corresponding author upon reasonable request.
