# Supplementary material for: Outcomes of CD19 CAR T in Transformed Indolent Lymphoma Compared to De Novo Aggressive Large B‐Cell Lymphoma
Source: Am J Hematol. 2024 Dec 23;100(2):236–48. doi: 10.1002/ajh.27548 (PMC11705210; doi:10.1002/ajh.27548)

**Supplemental Table 1. Other bridging therapy given**

| **Characteristic** | **Overall^1^** | **de novo^1^** | **tiNHL^1^** |
| --- | --- | --- | --- |
| **Other bridging therapy given** |  |  |  |
| Polatuzumab +/- rituximab +/- bendamustine | 43 (41) | 29 (40) | 14 (44) |
| BTK inhibitor based treatment | 18 (17) | 12 (17) | 6 (19) |
| Lenalidomide-based treatment | 10 (10) | 9 (13) | 1 (3) |
| CD20 mAb +/- steroid | 6 (6) | 3 (4) | 3 (9) |
| BTK inhibitor plus lenalidomide | 5 (5) | 3 (4) | 2 (6) |
| PD-1 mAb based treatment | 5 (5) | 5 (7) | 0 (0) |
| Intrathecal chemotherapy | 3 (3) | 3 (4) | 0 (0) |
| Loncastuximab | 2 (2) | 1 (1) | 1 (3) |
| Other | 12 (12) | 7 (10) | 5 (16) |
| *^1^n (%)* | | | |

Abbreviations: BTK- Bruton tyrosine kinase, mAB- monoclonal antibody, PD-1- programmed cell death protein 1

**Supplemental Table 2. Safety profile by indolent lymphoma in the tiNHL cohort (n=338)**

| **Characteristic** | **Overall N=338^1^** | **FL n=284^1^** | **MZL n=41^1^** | **WM n=13^1^** |
| --- | --- | --- | --- | --- |
| **CRS Any grade** | 267 (79) | 229 (81) | 29 (71) | 9 (69) |
| **Days between infusion and first CRS onset** | 3 (0 - 53) | 3 (0 - 15) | 4 (0 - 53) | 1 (0 - 6) |
| **CRS onset within the first 2 days post CAR T infusion** | 120 (36) | 103 (36) | 12 (29) | 5 (38) |
| **CRS ≥ Grade 3** | 24 (7) | 18 (6) | 6 (15) | 0 (0) |
| **Maximum CRS grade (Per ASTCT consensus grading)** |  |  |  |  |
| Grade 1 | 134 (40) | 119 (42) | 11 (27) | 4 (31) |
| Grade 2 | 109 (32) | 92 (32) | 12 (29) | 5 (38) |
| Grade 3 | 15 (4) | 10 (4) | 5 (12) | 0 (0) |
| Grade 4 | 8 (2) | 7 (2) | 1 (2) | 0 (0) |
| Grade 5 | 1 (0) | 1 (0) | 0 (0) | 0 (0) |
| No CRS | 71 (21) | 55 (19) | 12 (29) | 4 (31) |
| **Tocilizumab given for CRS** | 170 (50) | 142 (50) | 22 (54) | 6 (46) |
| **Glucocorticoids were given for CRS** | 88 (26) | 74 (26) | 12 (29) | 2 (15) |
| **ICANS Any grade** | 141 (42) | 117 (41) | 18 (44) | 6 (46) |
| **Days between infusion and first ICANS onset** | 6 (0 - 38) | 6 (0 - 38) | 6 (0 - 19) | 6 (1 - 13) |
| **ICANS ≥ Grade 3** | 70 (21) | 58 (20) | 9 (22) | 3 (23) |
| **Maximum ICANS grade (Per ASTCT consensus grading)** |  |  |  |  |
| Grade 1 | 26 (8) | 22 (8) | 4 (10) | 0 (0) |
| Grade 2 | 45 (13) | 37 (13) | 5 (12) | 3 (23) |
| Grade 3 | 57 (17) | 47 (17) | 8 (20) | 2 (15) |
| Grade 4 | 13 (4) | 11 (4) | 1 (2) | 1 (8) |
| No ICANS | 197 (58) | 167 (59) | 23 (56) | 7 (54) |
| **Glucocorticoids were given for ICANS** | 120 (36) | 99 (35) | 15 (37) | 6 (46) |
| **CAR T toxicity related ICU stay within the first 30 days post infusion** | 39 (12) | 30 (11) | 7 (17) | 2 (15) |
| *^1^n (%); Median (Range)* | | | | |

Abbreviations: CRS- cytokine release syndrome, ICANS- immune effector cell-associated neurotoxicity syndrome

**Supplemental Table 3. Disease response post-CAR T**

| **Characteristic** | **Overall N=1182** | **de novo n=844** | **tiNHL n=338** | **p-value^2^** |
| --- | --- | --- | --- | --- |
| **Best response** |  |  |  |  |
| Complete response | 704 (62) ^1^ | 485 (59) | 219 (67) |  |
| Partial response | 227 (20) | 174 (21) | 53 (16) |  |
| Stable disease | 19 (2) | 12 (1) | 7 (2) |  |
| Progressive disease | 194 (17) | 146 (18) | 48 (15) |  |
| Not assessed/Unknown | 38 | 27 | 11 |  |
| **Among patients who had disease response assessed (n=1144)** | | | |  |
| Overall response^3^ rate | 81% | 81% | 83% | 0.3 |
| Complete response rate | 62% | 59% | 67% | 0.017 |
| **Among patients who achieved overall response^3^ (n=931)** | | | |  |
| Time from CAR T infusion to first documented response (months) | 1.0 (0.5 – 9.3) | 1.0 (0.6 – 9.3) | 1.0 (0.5 – 4.1) | 0.3 |
| Time from CAR T infusion to best response (months) | 1.0 (0.5 – 22.3) | 1.0 (0.6 – 22.3) | 1.0 (0.5 – 12.2) | 0.8 |
| Median duration of response^4^ (months) | 21.2 (95%CI: 16.2 – 25.9) | 20.0 (95%CI: 12.1 – 25.9) | 23.6 (95%CI: 15.1 – 54.8) | 0.28 |
| **Among patients who achieved CR (n=704)** | | | |  |
| Time from CAR T infusion to complete response (months) | 1.0 (0.5 – 22.3) | 1.0 (0.6 – 22.3) | 1.0 (0.5 – 12.2) | 0.14 |
| Median duration of complete response^4^ (months) | 39.6 (95%CI: 29.6 – 48.3) | 36.3 (95%CI: 27.8 – 44.1) | 48.3 (95%CI: 23.6 – Not reached) | 0.81 |
| ^1^n (%); Median (range). ^2^Pearson’s Chi-squared test for response; Wilcoxon rank sum test for time to response; Log-rank test for duration of response. ^3^Overall response: Achieved CR or PR. ^4^Duration of response based on Kaplan-Meier estimates. | | | | |

**Supplemental Table 4. Progression/relapse and vital status**

| **Characteristic** | **Overall N=1182^1^** | **de novo n=844^1^** | **tiNHL n=338^1^** |
| --- | --- | --- | --- |
| **Disease progression/relapse** | 585 (52) | 425 (53) | 160 (50) |
| Not assessed/Unknown | 52 | 37 | 15 |
| **Was a biopsy done at the time of progression/relapse?** |  |  | 82 (51) |
| **Histology at the time of relapse/progression**  *Median (range) time from infusion to progression/relapse (months)* |  |  |  |
| DLBCL/HGBCL |  |  | 72 (88)  *3.1 (0.3 – 24.0)* |
| Indolent lymphoma |  |  | 8 (10)  *10.2 (1.6 – 22.2)* |
| Both |  |  | 2 (2)  *5.0 (3.0 – 7.0)* |
| **Vital status at last contact** |  |  |  |
| Alive | 645 (55) | 451 (53) | 194 (57) |
| Dead | 537 (45) | 393 (47) | 144 (43) |
| ***CAR T therapy related death*** |  |  |  |
| *Yes* | *45 (8)* | *31 (8)* | *14 (10)* |
| *No* | *488 (91)* | *360 (92)* | *128 (89)* |
| *Unknown* | *4 (1)* | *2 (1)* | *2 (1)* |
| ^1^n (%); ^2^Other causes of death include cardiac failure, respiratory failure, GI hemorrhage, multiple organ failure, bronchiolitis obliterans, liver failure, acute kidney injury, shock, stroke, transitioned to comfort measures only, vascular disease, and hemorrhagic complications. | | | |

Abbreviations: DLBCL- diffuse large B-cell lymphoma, HGBCL- high grade B-cell lymphoma, CRS- cytokine release syndrome, ICANS- immune effector cell-associated neurotoxicity syndrome, IEC-HS- immune effector associated hemophagocytic lymphohistiocytosis-like syndrome.

**Supplemental Table 5. Post-hoc analysis: Multivariable Cox proportional hazards model for Progression/relapse Free Survival (de novo vs. tFL)**

| **Variable** | **Group** | **N^1^** | **Number of events** | **Adjusted HR (95%CI)** | **Forest Plot: Adjusted HR (95% CI)** | **p-value** |
| --- | --- | --- | --- | --- | --- | --- |
| Cohort | de novo | 769 | 483 | 1 (Ref.) | 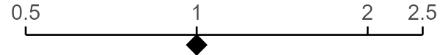 |  |
|  | tFL | 260 | 148 | 0.81 (0.66, 1) | 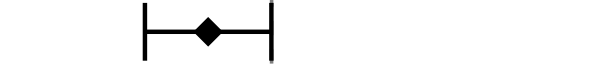 | 0.049 |
| Age (yrs) at infusion | Every additional 1 year older | | | 1 (0.99, 1) | 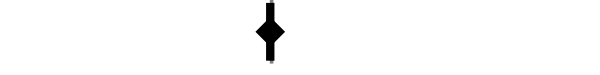 | 0.183 |
| Disease stage (Ann Arbor) prior to CAR T | I-II | 198 | 101 | 1 (Ref.) | 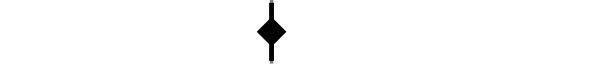 |  |
|  | III-IV | 831 | 530 | 1.4 (1.1, 1.8) | 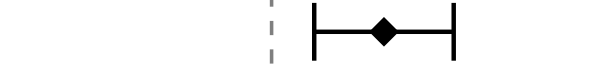 | 0.002 |
| ECOG Performance score prior to CAR T | 0-1 | 895 | 542 | 1 (Ref.) | 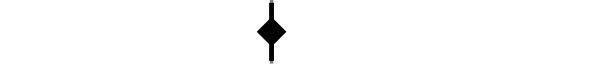 |  |
|  | 2-4 | 134 | 89 | 0.98 (0.78, 1.2) | 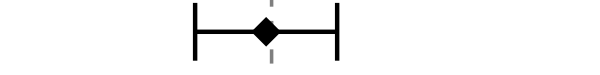 | 0.88 |
| LDH > ULN prior to CAR T (at leukapheresis) | No | 427 | 214 | 1 (Ref.) | 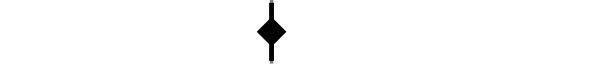 |  |
|  | Yes | 602 | 417 | 1.7 (1.4, 2) | 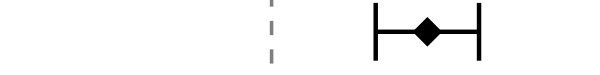 | <0.001 |
| >1 extranodal site prior to CAR T | No | 620 | 364 | 1 (Ref.) | 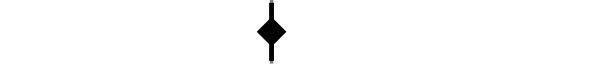 |  |
|  | Yes | 409 | 267 | 1 (0.86, 1.2) | 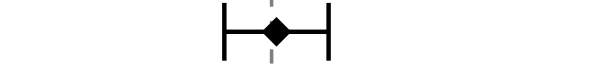 | 0.854 |
| Prior lines of therapy before CAR T infusion (not including bridging) ≥ 3 | No | 458 | 256 | 1 (Ref.) | 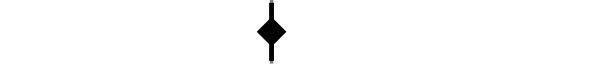 |  |
|  | Yes | 571 | 375 | 1.2 (1, 1.4) | 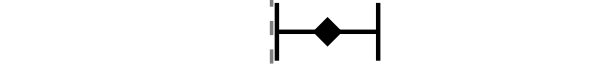 | 0.03 |
| Prior bendamustine use^2^ timing relative to CAR T infusion | None | 859 | 520 | 1 (Ref.) | 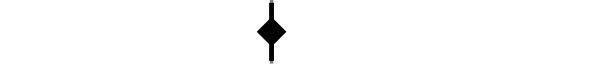 |  |
|  | Remote (>12 months) | 79 | 41 | 0.82 (0.58, 1.2) | 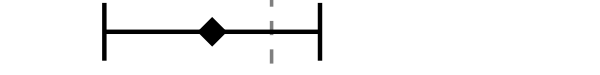 | 0.28 |
|  | Recent (within 12 months) | 91 | 70 | 1.4 (1.1, 1.8) | 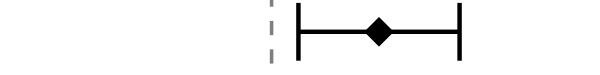 | 0.009 |
| Evidence of CNS involvement of aLBCL prior to CAR T | No | 927 | 550 | 1 (Ref.) | 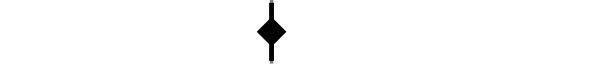 |  |
|  | Yes | 102 | 81 | 1.3 (1, 1.7) | 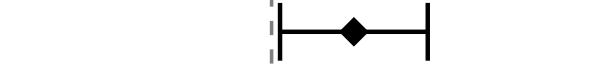 | 0.029 |
| Receipt of bridging therapy | No | 511 | 281 | 1 (Ref.) | 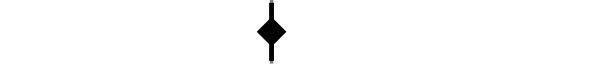 |  |
|  | Yes | 518 | 350 | 1.4 (1.2, 1.7) | 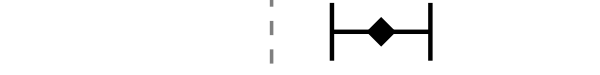 | <0.001 |
| ^1^ As post-hoc analysis, we excluded 54 patients with tMZL or tWM from the 1182 overall patients to explore the difference in PFS between the de novo cohort and patients with tFL. 99 out of 1128 patients had unknown disease stage, ECOG, LDH, extranodal site, and/or CNS involvement of aLBCL prior to CAR T thus were further excluded from the multivariable model. ^2^ Prior bendamustine use: Before apheresis (not including LD or bridging therapy). | | | | | | |

Abbreviations: CAR T: chimeric antigen receptor T-cell; ECOG: Eastern Cooperative Oncology Group; LDH: lactate dehydrogenase; ULN: upper limit of normal; CNS: central nervous system.

**Supplemental Figure 1. Best response post CAR T**


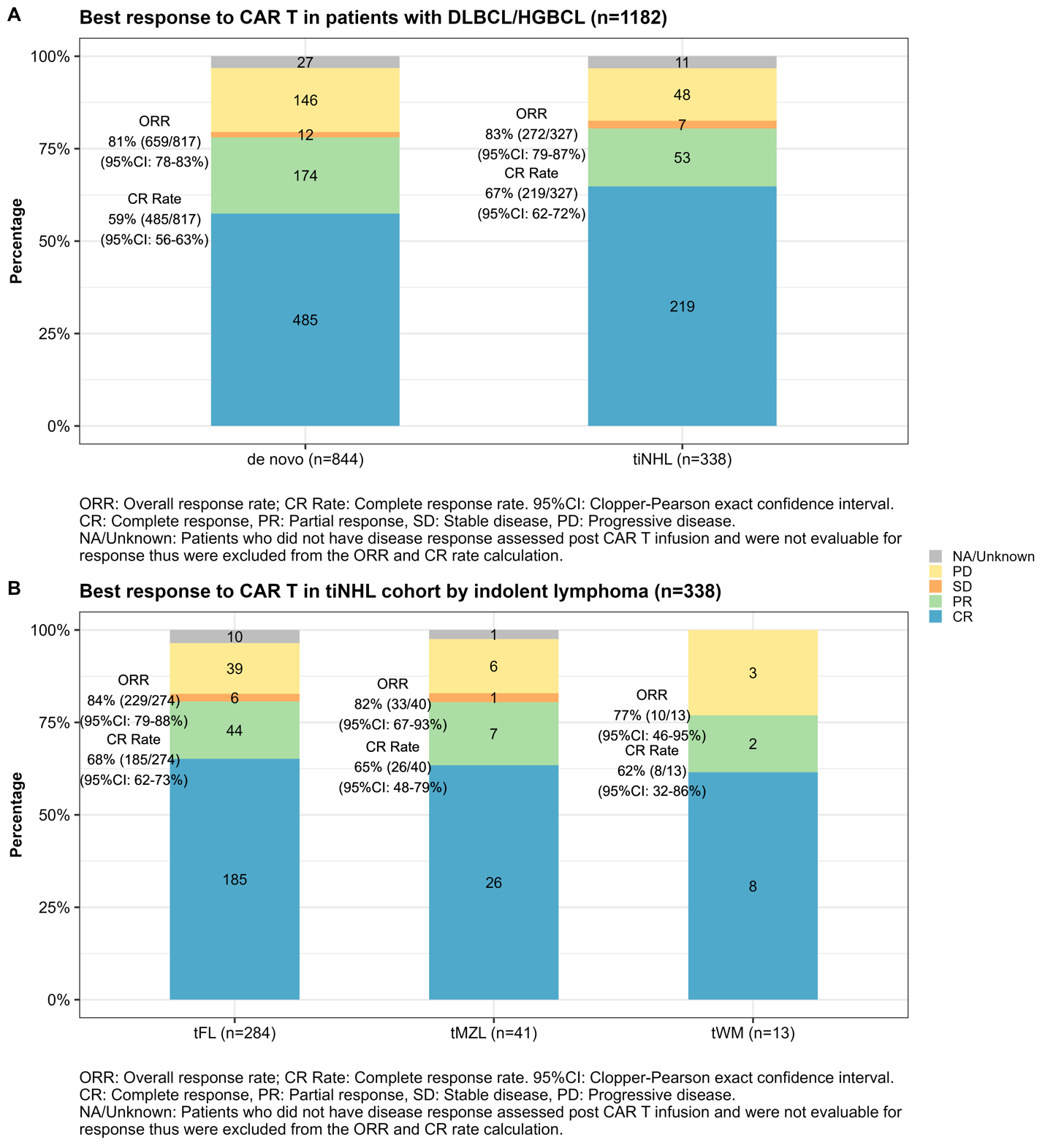


**Supplemental Figure 2. PFS post CAR T infusion by (A) timing of prior bendamustine use (before apheresis not including LD or bridging therapy), and (B) receipt of bridging therapy**


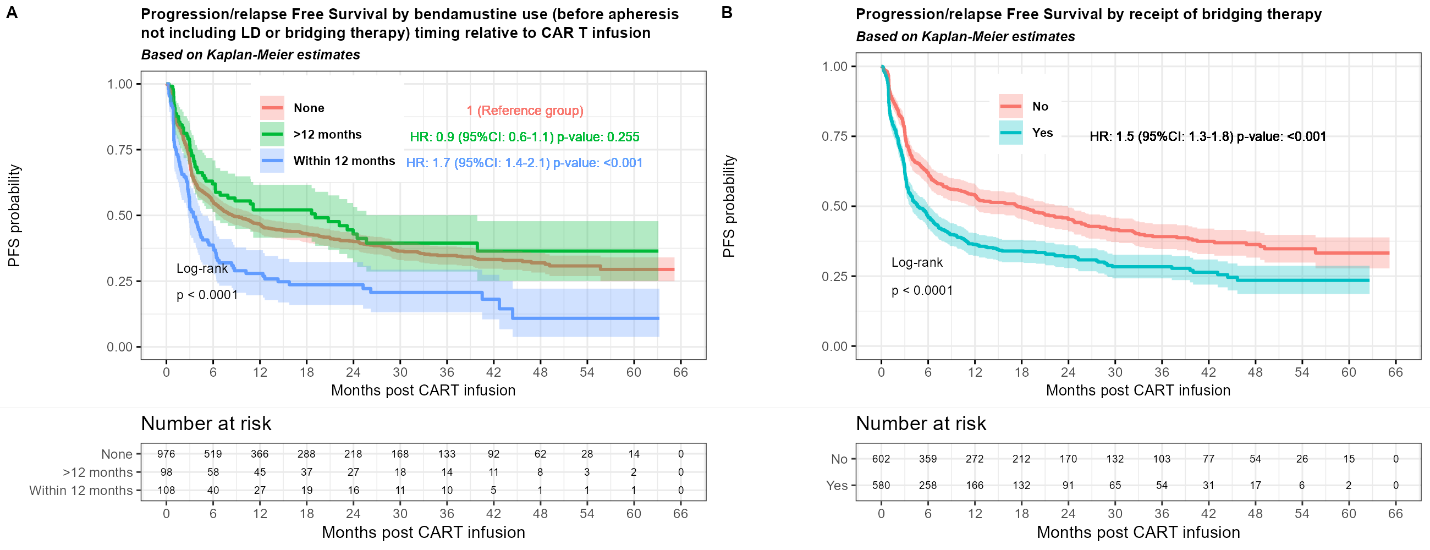


**Supplemental Figure 3. In the tiNHL cohort, PFS post CAR T infusion by (A) concurrent/sequential, (B) prior treatment for indolent disease before transformation, and (C) type of indolent lymphoma**


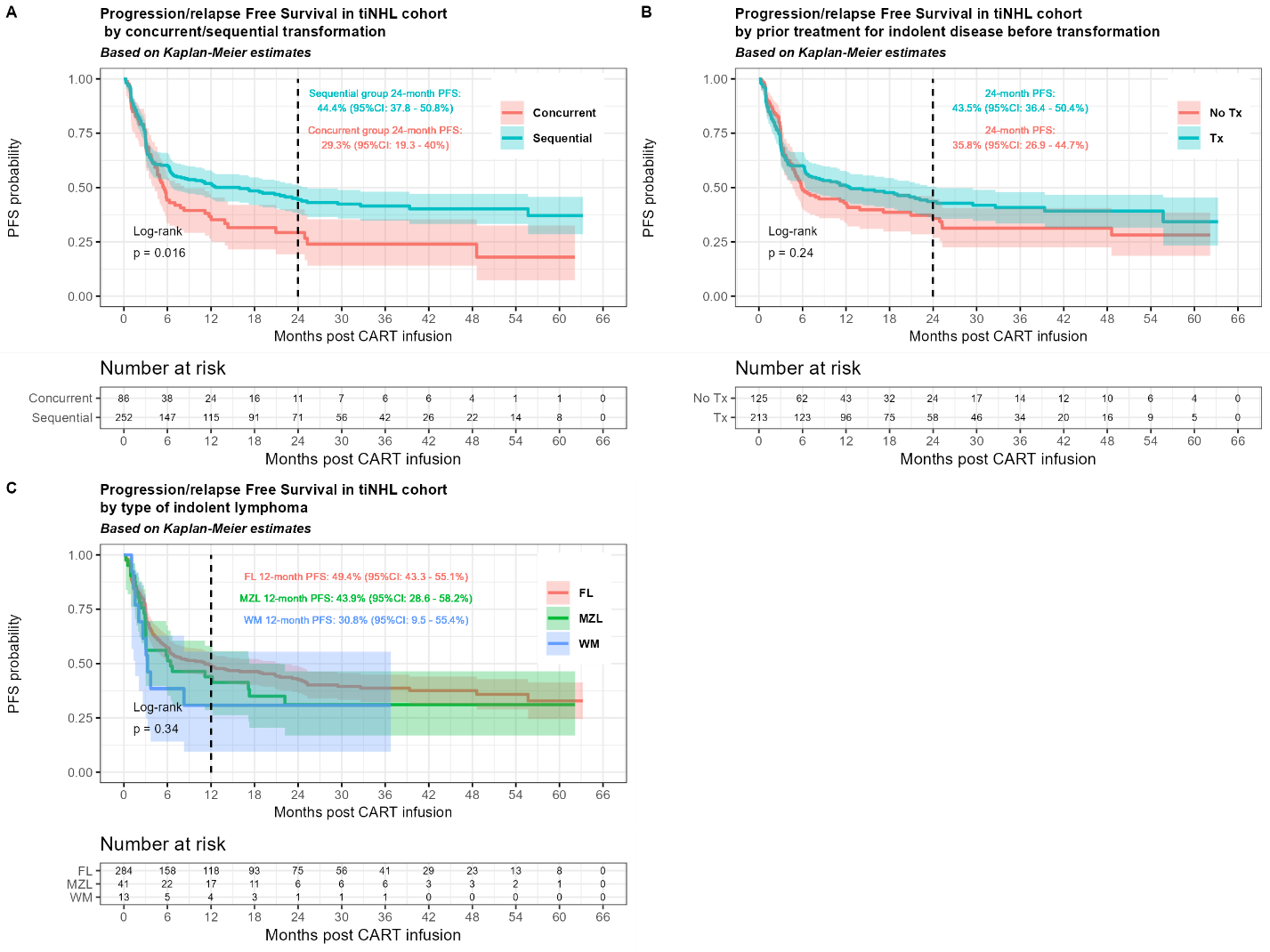


**Supplemental Figure 4. Kinetics of IgM paraprotein changes in Waldenstrom patients post CAR T**


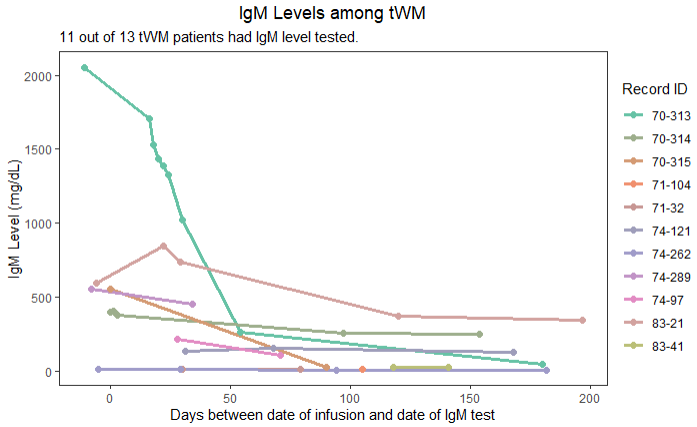

Supplement: Supplementary file 1 — Data S1. Supporting Information. [file AJH-100-236-s001.docx]
